# Supplementary material for: Tumor-penetrating therapy for β5 integrin-rich pancreas cancer
Source: Nat Commun. 2021 Mar 9;12:1541. doi: 10.1038/s41467-021-21858-1 (PMC7943581; doi:10.1038/s41467-021-21858-1)
Supplement: Supplementary file 1 — Supplementary Information [file 41467_2021_21858_MOESM1_ESM.docx]

# Supplementary Materials

Fig. S1. iRGD spreading in stroma-rich breast tumors

Fig. S2. Expression of iRGD receptors in pancreatic and breast CAFs

Fig. S3. iRGD entry into CAFs and tumor cells in 2D culture systems

Fig. S4. CAFs enhance iRGD entry into HeLa cells

Fig. S5. 3D-Culture upregulates αvβ5 expression and iRGD-AgNP uptake in hPC1356 PDAC cells

Fig. S6. The effect of soluble factors and EVs in CAF CM on αvβ5 expression on tumor cells

Fig. S7. The effect of CAF CM on αvβ5 mRNA expression and transfer in tumor cells

Fig. S8. The effect of TGF-β on the cell surface expression of αvβ5 and NRP-1 in tumor cells.

Fig. S9. CAF CM upregulates TGF-β and its target genes in PDAC cells.

Fig. S10. β5 integrin knockout (KO) clones generated in LM-PmC cells

Fig. S11. β5 integrin-dependent spreading of iRGD in PDAC

Fig. S12. iRGD co-injection potentiates accumulation of co-administered dextran in KPC derived mouse tumors

Fig. S13. Abundant expression of αvβ5 integrin in the PDAC of KPC mice

Fig. S14. iRGD co-injection increases Evans Blue entry into tumors in KPC mice

Fig. S15. Survival curve of KPC mice treated with iRGD alone

Fig. S16. Enhanced apoptosis of PDAC cells in KPC mice treated with iRGD + gemcitabine combination therapy

Fig. S17. Flow cytometry gating strategy

# Table S1. Primer sequences for the gene expression analysis performed by QPCR

**
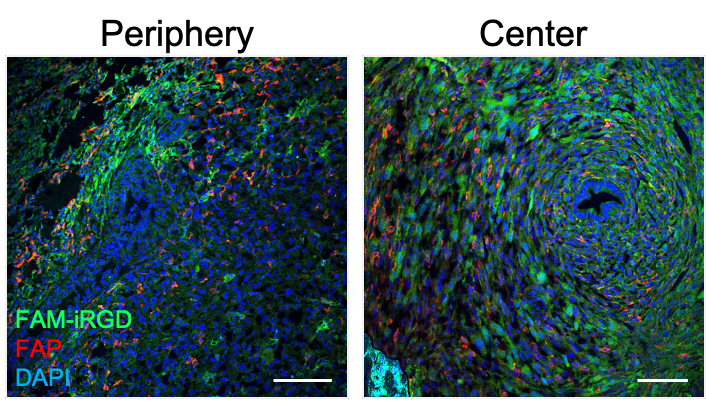
**

**Fig. S1 - iRGD spreading in stroma-rich breast tumors.** Representative confocal micrographs showing the penetration of intravenously injected FAM-iRGD (green) into orthotopic breast tumors in mice generated with MCF10CA1a human breast cancer cells. The periphery (left) and center (right) of a tumor are shown. Red, FAP; blue, DAPI. Scale bars, 50 µm. (*n* = 3 mice).


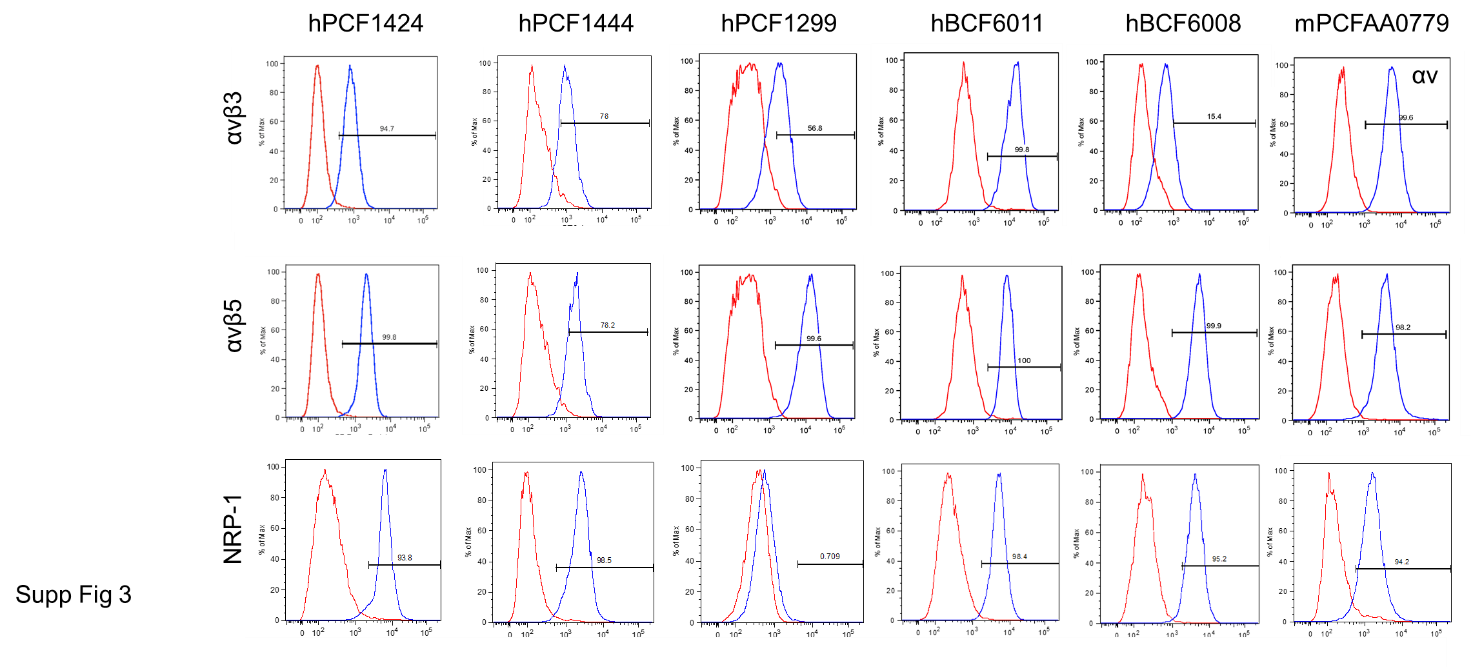


# Fig. S2 - Expression of iRGD receptors in pancreatic and breast CAFs. Expression of αvβ3, αvβ5 and NRP-1 in CAFs analyzed by flow cytometry (blue). Red, isotype controls. Human PDAC (hPCF1424, hPCF1444, hPCF1299), breast (hBCF6011, hBCF6008), and mouse PDAC (mPCFAA0779) CAFs were analyzed. For mPCFAA0779 CAFs, cell surface expression of αv integrins instead of αvβ3 is shown given the unavailability of an adequate anti-mouse αvβ3 antibody.


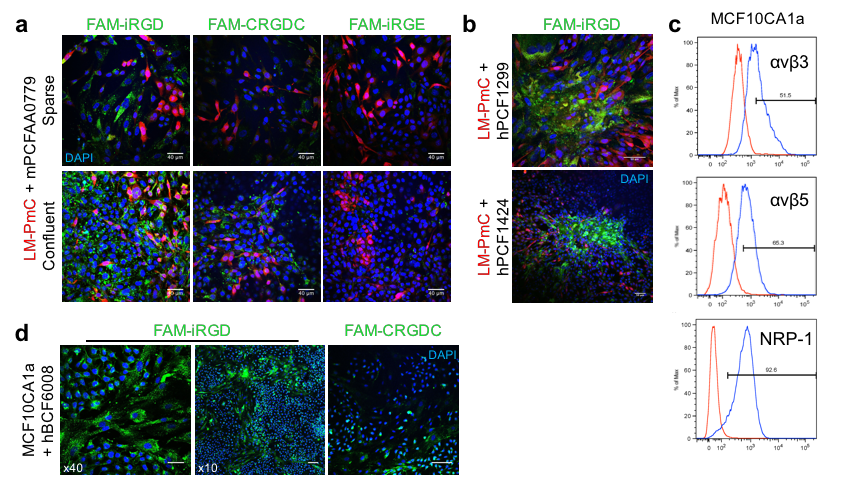


**Fig. S3 - iRGD entry into CAFs and tumor cells in 2D culture systems. a** Confocal micrographs of LM-PmC PDAC cells (red) co-cultured with mPCFAA0779 PDAC CAFs in the presence of peptides (green). The peptides were FAM-iRGD, FAM-CRGDC (an RGD peptide without a CendR motif), and FAM-iRGE (an RGE variant of iRGD). Upper panels, sparsely grown; lower panels, confluent. Blue, DAPI. Scale bars, 40 µm. **b** Confocal micrographs of LM-PmC cells (red) co-cultured with hPCF1299 or hPCF1424 PDAC CAFs in the presence of FAM-RGD (green). Blue, DAPI. Scale bars, 50 µm. **c** Cell surface expression of αvβ3 and

αvβ5 integrins and NRP-1 in MCF10CA1a human breast cancer cells analyzed by flow cytometry (blue). Red, isotype controls. **d** Confocal micrographs of MCF10CA1a cells co- cultured with hBCF6008 CAFs in the presence of FAM-RGD (green) or FAM-CRGDC (green). Blue, DAPI. Scale bars, 50 µm (40 µm in the left panel). (*n* = 3 independent experiments for **(a)**, **(b)** and **(d)**).


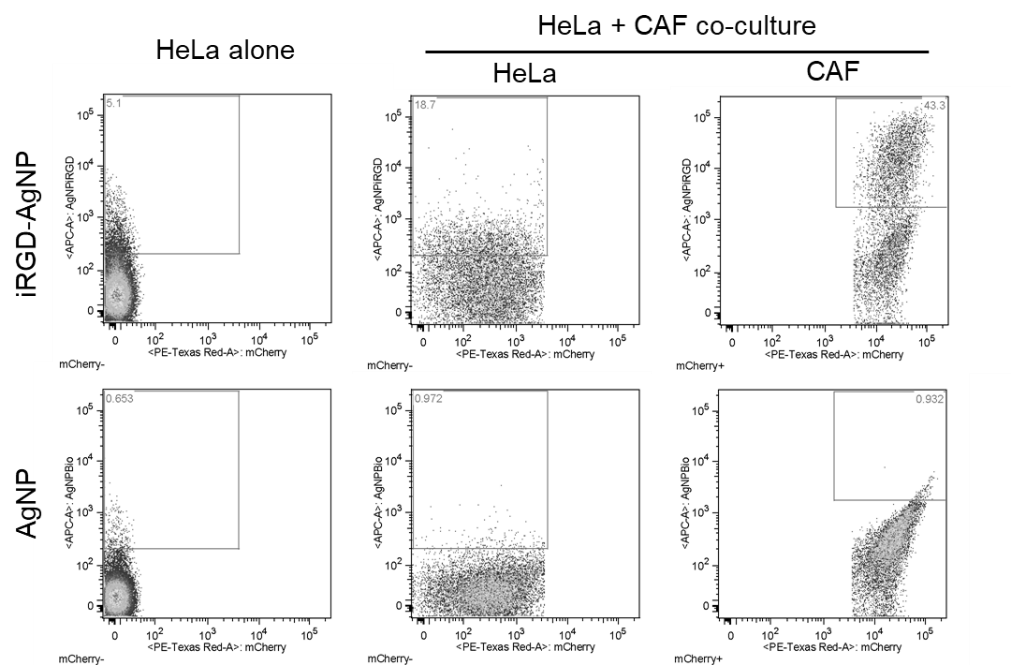


**Fig. S4 - CAFs enhance iRGD entry into HeLa cells.** Flow cytometry analysis showing entry of iRGD-AgNPs or control AgNPs into spheroids made of HeLa cells alone or HeLa cells mixed with mCherry-labeled hPCF1424 CAFs. Note that iRGD-AgNPs entered HeLa cells more efficiently in the presence of CAFs. The presence of CAFs had minimal effect on the entry of control AgNPs. Representative data of 3 independent experiments.


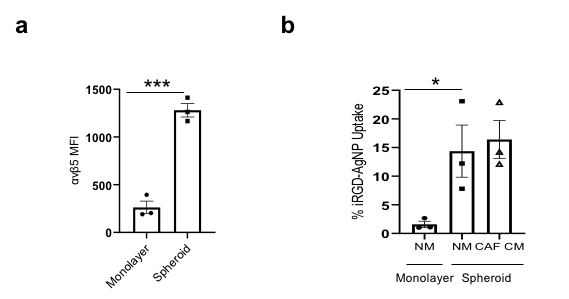


**Fig. S5 - 3D-Culture upregulates αvβ5** **expression and iRGD-AgNP uptake in**

**hPC1356 PDAC cells. a** Cell surface expression of αvβ5 integrin in hPC1356 cells grown as monolayers or spheroids. Median fluorescence intensity (MFI) was measured by flow cytometry (data representative of 3 biological experiments). Two-tailed unpaired student’s t-test; *p* = 0.0005. **b** The proportion of hPC1356 cells that took up iRGD-AgNPs. The cells were cultured as monolayers or spheroids in either normal media (NM) or CAF CM. *n* = 3 independent experiments. One-way ANOVA; *p* = 0.0322 (monolayer vs spheroids cultured in NM), *p* = 0.8933 (spheroids cultured in NM vs CM). Error bars, SEM; **p* < 0.05; ****p* < 0.001. Source data provided in Source Data.

**
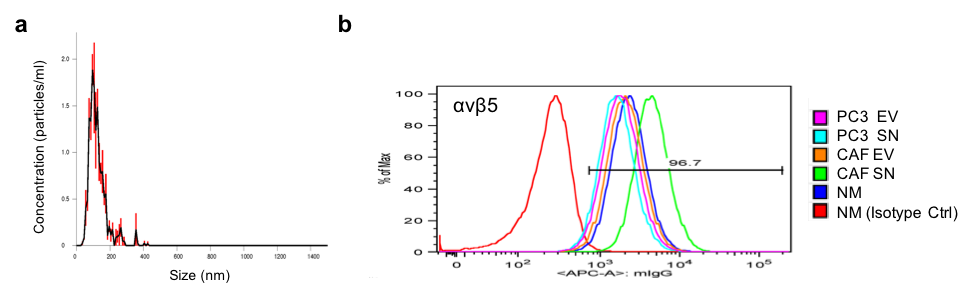
**

**Fig. S6 - The effect of soluble factors and EVs in CAF CM on αvβ5 expression on tumor cells. a** Nanoparticle tracking analysis showing the size distribution of the EVs isolated from the CM of hPCF1424 CAFs. **b** Expression of αvβ5 integrin in PC3 cells incubated with NM or the EV or EV-depleted supernatant (SN) fractions prepared from CM of PC3 cells or hPCF1424 CAFs. Red, isotype control. Data representative of 3 independent experiments.

**
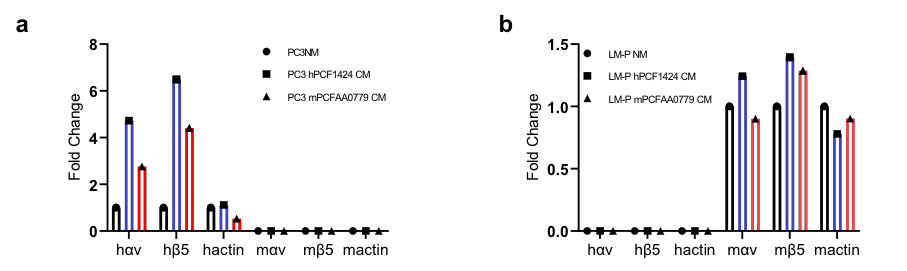
Fig. S7** - **The effect of CAF CM on αvβ5 mRNA expression and transfer in tumor cells.**

**a** mRNA expression of human (hITGαv) and mouse (mITGαv) αv integrin, human (hITGβ5) and mouse (mITGβ5) β5 integrin, and human (hactin) and mouse (mactin) β-actin analyzed by qPCR in PC3 human tumor cells incubated with normal media (NM) or CM from hPCF1424 human PDAC CAFs or mPCFAA0779 mouse PDAC CAFs. Note that an increase in human αvβ5 mRNAs was noted upon treatment with both CAF CM preparations. Mouse αvβ5 mRNAs were not detected in the human tumor cells upon treatment with mouse CAF CM. **b** Expression of mouse and human mRNAs of αv and β5 integrin subunits and β-actin analyzed by qPCR in LM-P mouse tumor cells incubated with NM or CM from hPCF1424 or mPCFAA0779 CAFs. Human αvβ5 mRNAs were not detected in the mouse tumor cells upon treatment with human CAF CM. Data representative of 3 independent experiments. Source data provided in Source Data.


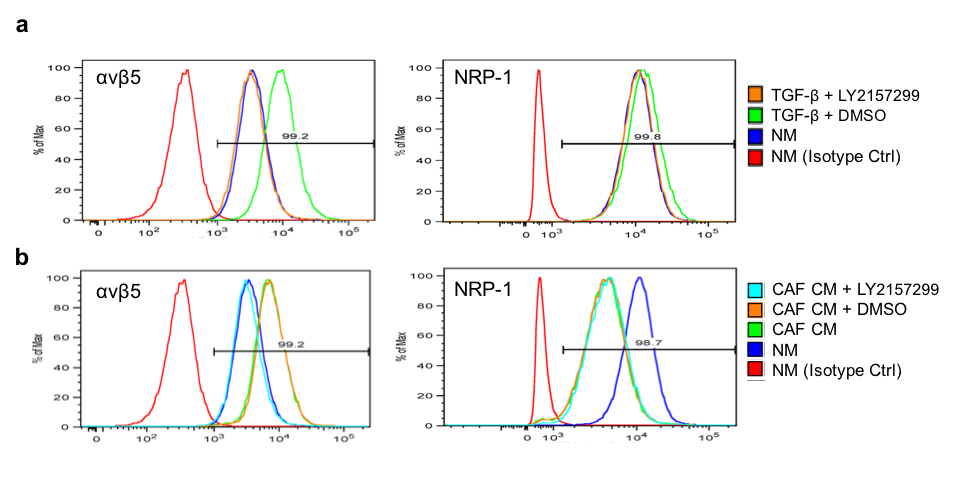


**Fig. S8 - The effect of TGF-β on the cell surface expression of αvβ5 and NRP-1 in tumor cells. a** Cell surface expression of αvβ5 integrin (left panel) and NRP-1 (right panel) in PC3 cells cultured in hPCF1424 CAF CM alone or in the CM with a TGF-β-specific inhibitor LY2157299. DMSO was the vehicle control for the inhibitor. Some cells were cultured in NM as a reference for the CAF CM. **b** Cell surface expression of αvβ5 integrin (left panel) and NRP-1 (right panel) in PC3 cells cultured in normal media (NM) and in the presence of exogenous TGF-

β with a TGF-β-specific inhibitor LY2157299 or vehicle alone (DMSO). *n* = 4 independent experiments. Refer to Figure 4e and f for quantitative data.

**
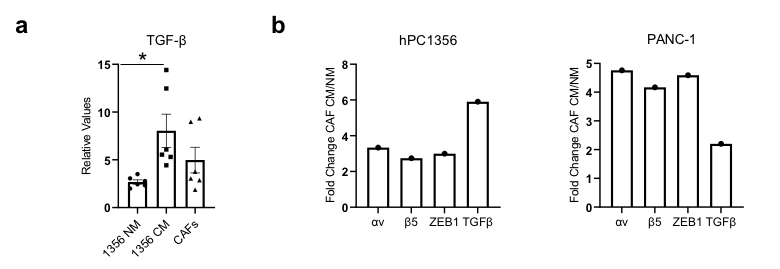
**

**Fig. S9 - CAF CM upregulates TGF-β and its target genes in PDAC cells. a** Expression of TGF-β mRNA in hPC1356 PDAC cells cultured in normal media (NM) or CAF CM and in hPCF1424 CAFs cultured in NM. Relative values normalized to cyclophilin are shown. *n* = 6 independent experiments, error bars, SEM. Two-tailed unpaired student’s t-test; **p* = 0.027 (1356 NM vs CM). **b** qPCR data showing αv and β5 integrin, ZEB1, and TGF-β mRNA expression in hPC1356 (left panel) and PANC-1 (right panel) PDAC cells cultured in NM or CAF CM. Fold over NM normalized against cyclophilin is shown. Representative data from 4 independent experiments. Source data provided in Source Data.


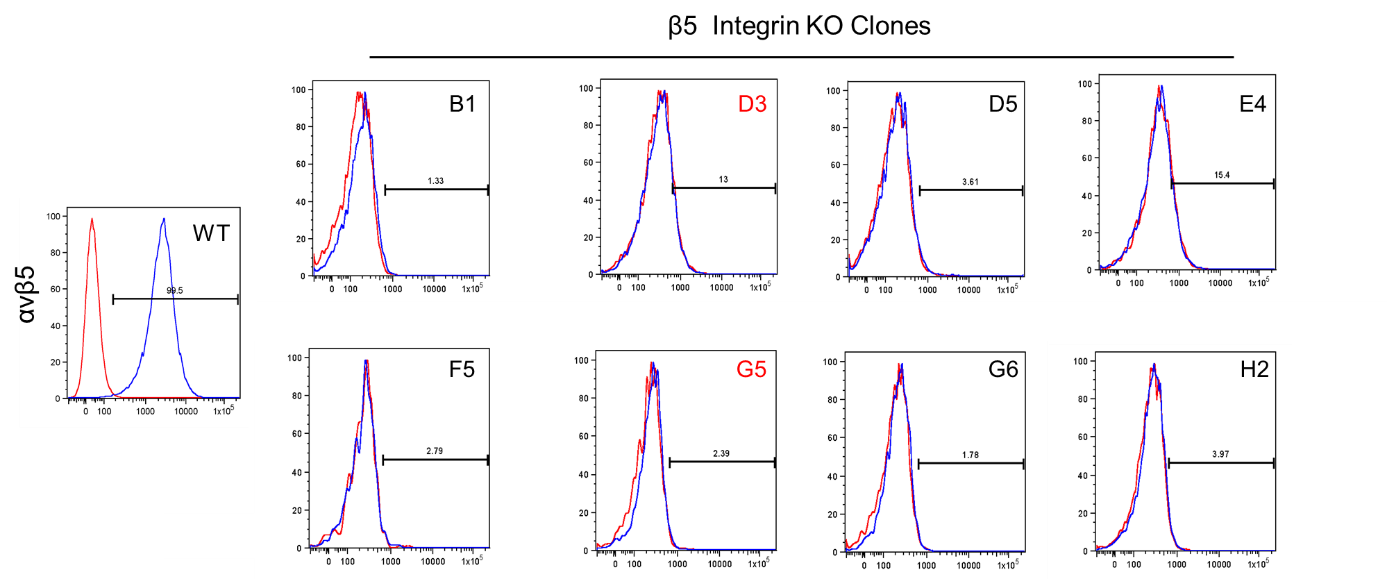


**Fig. S10 - β5 integrin knockout (KO) clones generated in LM-PmC cells.** Expression of αvβ5 integrin (blue) in the original LM-PmC tumor cells (WT) or eight different β5 integrin KO clones generated with CRISPR. Red, isotype control.


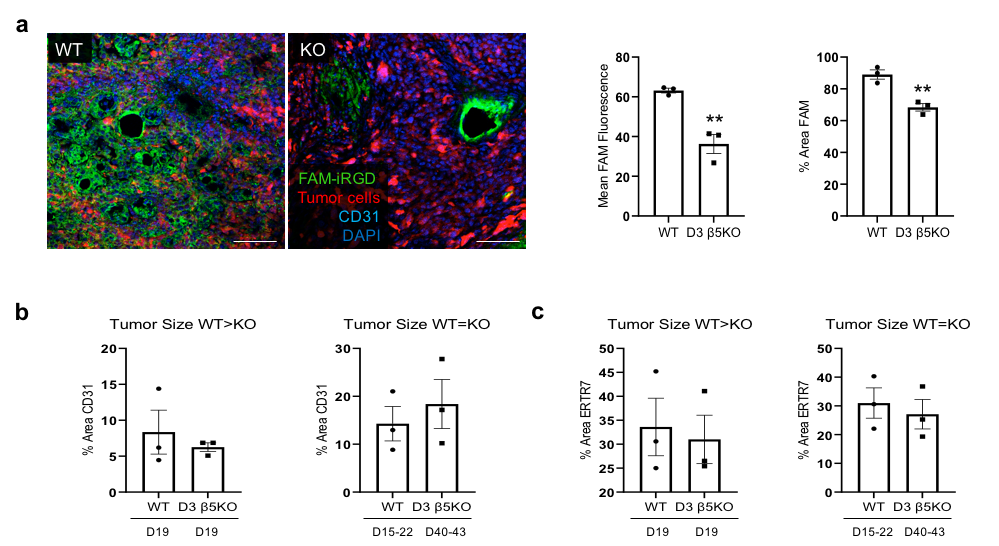


**Fig. S11 - β5 integrin-dependent spreading of iRGD in PDAC. a** Confocal micrographs showing spreading of intravenously delivered FAM-iRGD (green) in WT and β5 integrin KO LM-PmC tumors. Mice bearing orthotopic WT tumors 15-22 days after implantation and KO D3 clone tumors 40-43 days after implantation were used to match the sizes of the tumors. Red, tumor cells; blue, DAPI; scale bars, 50 μm. In the bar diagram, the mean FAM intensity was measured to quantify the amount of iRGD that homed to the tumor (left diagram) and % area positive for FAM was used to determine the degree of iRGD spreading (right diagram). *p* = 0.0055 (mean FAM), *p* = 0.0052 (% area FAM). **(b and c)** The % area positive for CD31 (b) and ERTR7 (c) in WT and β5 KO LM-PmC tumors are shown. Left panels, WT tumors from day 19 and β5 KO tumors from day 19 (refer to Fig. 5b); right panels, WT tumors from days 15-22 and β5 KO tumors from days 40-43 (refer to Fig. 5d). *n* = 3 mice per group. *p* = 0.54 (B: left panel), *p* = 0.55 (B: right panel), *p* = 0.757 (C: left panel), *p* = 0.625 (C: right panel). All error bars, SEM; two-tailed unpaired student’s t-test for all the panels; ***p* < 0.01. Source data provided in Source Data.


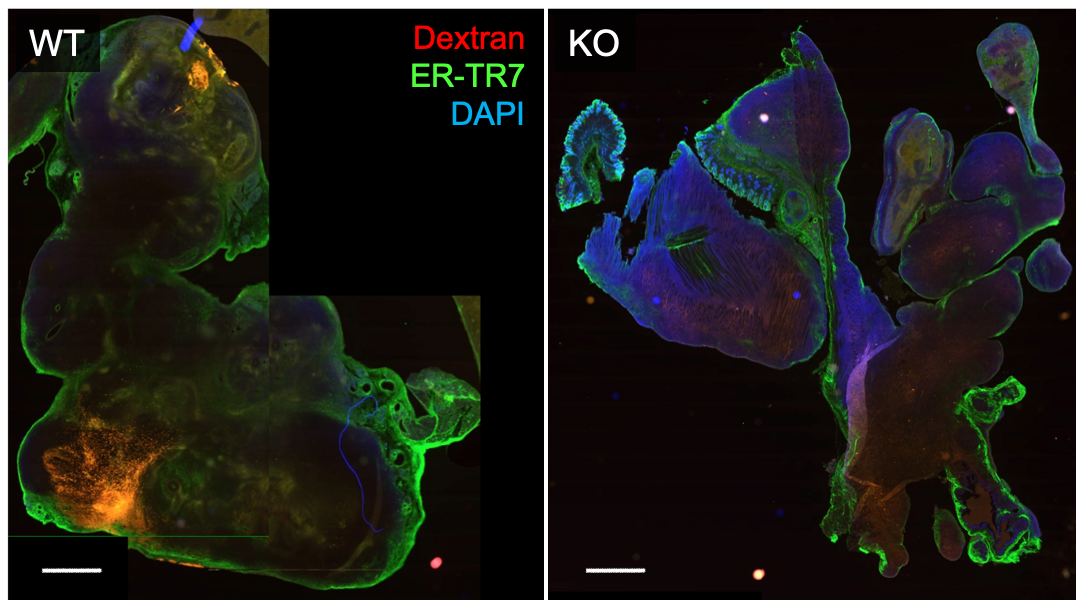


**Fig. S12 - iRGD co-injection enhances accumulation of co-administered dextran in KPC derived mouse tumors.** Representative immunofluorescent images showing the spreading of fluorescent dextran (red) in orthotopic PDAC created with β5 integrin-deficient LM-PmC cells (D3 clone, KO) or wild-type LM-PmC cells (WT) in mice. The dextran was co-injected with iRGD into the tail vein. Green, ER-TR7; blue, DAPI. Scale bars, 2 mm. Quantitative data shown in Fig 6A. *n* = 5 WT mice and 3 KO mice.


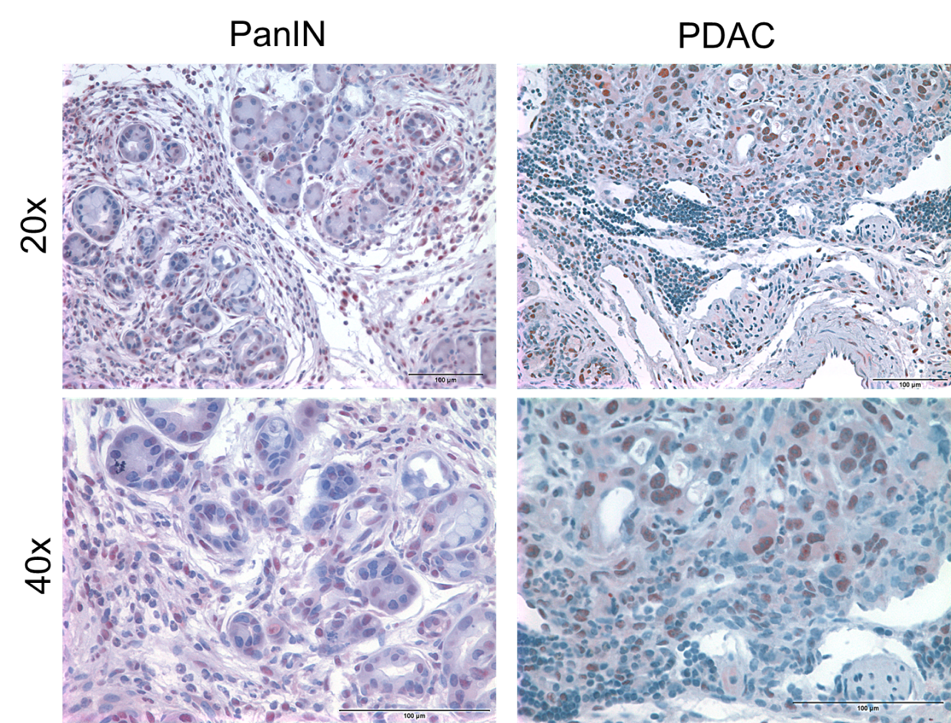


**Fig. S13 - Abundant expression of αvβ5 integrin in the PDAC of KPC mice.** *De novo* pancreatic tumors harvested from KPC mice (*n* = 3) were stained with an anti-αvβ5 integrin antibody and H&E. Areas containing PanINs and full-blown PDAC are shown in two different magnifications. Scale bars, 100 µm.


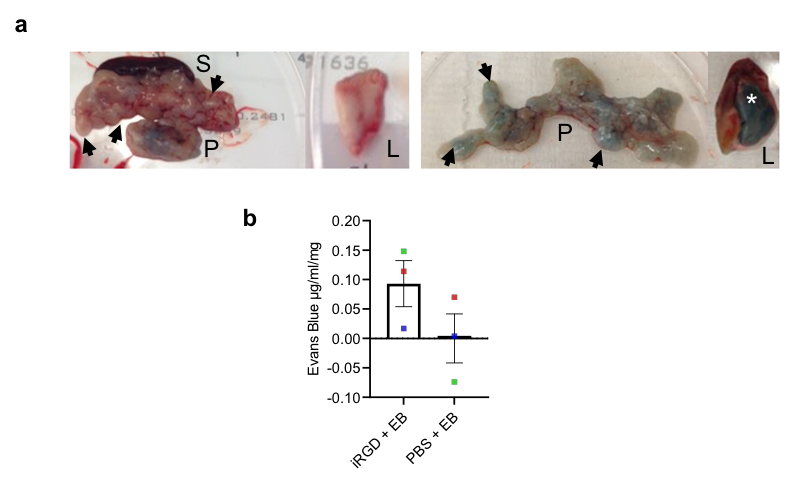


**Fig. S14 - iRGD co-injection increases Evans Blue entry into tumors in KPC mice. a** Images of the pancreas (P), spleen (S) and lung (L) collected from KPC mice injected with Evans Blue (EB) in combination with PBS or iRGD. Arrows point to some of the many tumor nodules, * labels a metastatic lesion in the lung with high EB uptake. Note the darker nodules in the iRGD group compared to the PBS group. **b** Quantification of EB in KPC mouse tumors normalized against wet tissue weight. Three independent paired experiments were performed as labeled in the bar diagram (each pair has the same color). Each experiment had one mouse injected with PBS + EB and one with iRGD + EB. Error bars, SEM; two-tailed paired student’s t-test, *p* = 0.2894. Source data provided in Source Data.


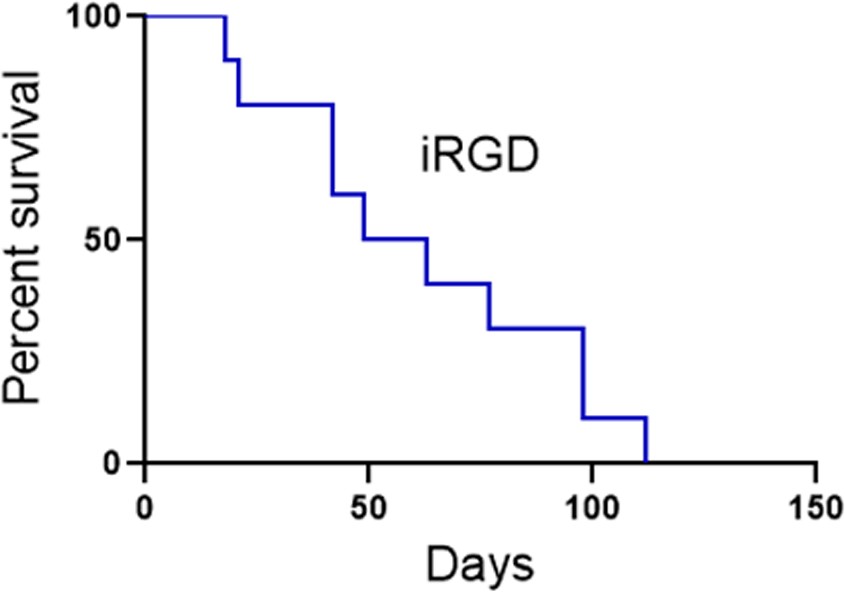


**Fig. S15 - Survival curve of KPC mice treated with iRGD alone.** iRGD alone did not provide any therapeutic benefit compared to historical survival data of untreated KPC mice (*n* = 10; median survival, 56 days) (*1*). Source data provided in Source Data.


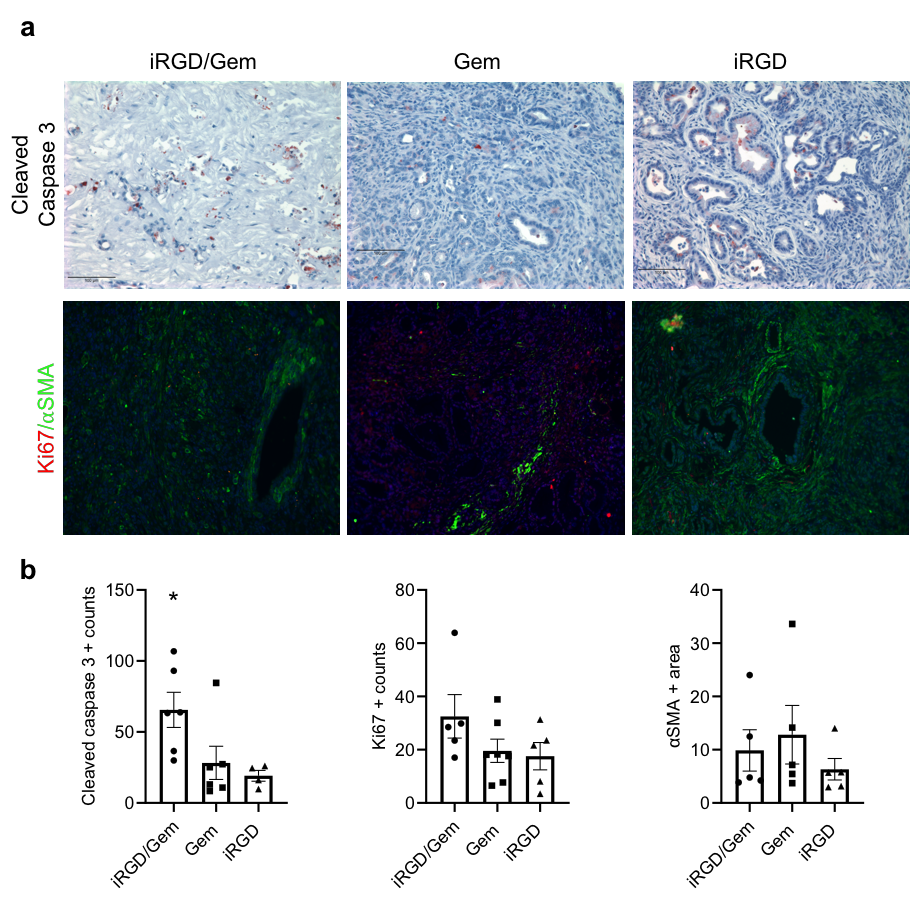


**Fig. S16 - Enhanced apoptosis of PDAC cells in KPC mice treated with iRGD + gemcitabine combination therapy. a** PDAC sections from KPC mice treated with iRGD + gemcitabine (Gem), Gem alone, or iRGD alone were stained for apoptotic cells using an anti-Cleaved caspase 3 antibody (brown, upper panels), proliferating cells using an anti-Ki67 antibody (red, lower panels), or activated fibroblasts using an anti-αSMA antibody (green, lower panels). Scale bars, 100 μm. **b** Cells positive for cleaved caspase 3, Ki67, or αSMA were counted in the sections stained in (a). Ten windows were randomly selected from at least 4 mice per group. Error bars, SEM. One-way ANOVA; *p* = 0.0279 (cleaved caspase 3), *p* = 0.203 (Ki67), *p* = 0.543 (αSMA). Source data provided in Source Data.

#
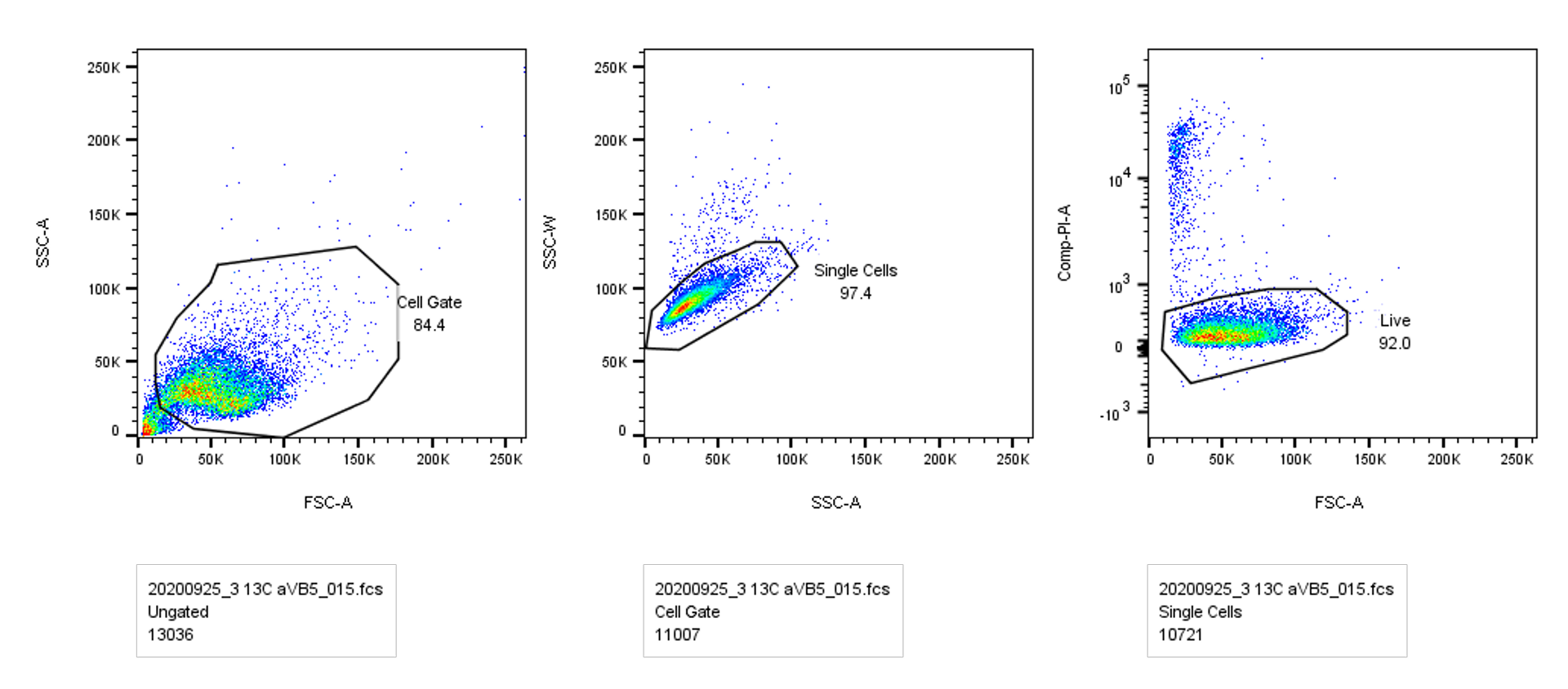


# Fig. S17 - Flow cytometry gating strategy. In order to exclude debris a cell gate was made in a FSA/SSC plot, then single cells were gated in a SSA/SSW plot, followed by live cell gating of cells negative for propidium iodide (PI) or DAPI.

| Gene | Species | Sequence |
| --- | --- | --- |
| ITGav | Mouse | Forward CCC GTT AAG TAA TAG TGT GGT CTC |
| ITGav | Mouse | Reverse GCA GCC AAA CAA AGG GAA TAG |
| ITGav | Human | Forward AAT CTT CCA ATT GAG GAT ATC AC |
| ITGav | Human | Reverse AAA ACA GCC AGT AGC AAC AAT |
| ITGb5 | Mouse | Forward GGA TCA GCC AGA AGA CCT TAA T |
| ITGb5 | Mouse | Reverse AAT CTT CAG ACC CTC ACA CTT C |
| ITGb5 | Human | Forward GGA GCC AGA GTG TGG AAA CA |
| ITGb5 | Human | Reverse GAA ACT TTG CAA ACT CCC TC |
| β-Actin | Human/Mouse | Forward GCT GTG CTA CGT CGC CCT G |
| β-Actin | Human/Mouse | Reverse GGA GGA GCT GGA AGC AGC C |
| Cyclophilin-A | Human/Mouse | Forward CTC GAA TAA GTT TGA CTT GTG TTT |
| Cyclophilin-A | Human/Mouse | Reverse CTA GGC ATG GGA GGG AAC A |
| TGF-β1 | Human | Forward GGA AAT TGA GGG CTT TCG CC |
| TGF-β1 | Human | Reverse CCG GTA GTG AAC CCG TTG AT |
| ZEB1 | Human | Forward GCC AAT AAG CAA ACG ATT CTC |
| ZEB1 | Human | Reverse TTT GGC TGG ATC ACT TTC AAG. |

# Table S1 - Primer sequences for the gene expression analysis performed by QPCR.

# Supplemental References

1. S. R. Hingorani, L. Wang, A. S. Multani, C. Combs, T. B. Deramaudt, R. H. Hruban, A. K. Rustgi, S. Chang, D. A. Tuveson, Trp53R172H and KrasG12D cooperate to promote chromosomal instability and widely metastatic pancreatic ductal adenocarcinoma in mice. *Cancer Cell* **7**, 469-483 (2005).
