# Supplementary material for: Tumor-penetrating therapy for β5 integrin-rich pancreas cancer
Source: Nat Commun. 2021 Mar 9;12:1541. doi: 10.1038/s41467-021-21858-1 (PMC7943581; doi:10.1038/s41467-021-21858-1)
Supplement: Supplementary file 3 — Reporting Summary [file 41467_2021_21858_MOESM3_ESM.pdf]

nature research

Corresponding author(s): Kazuki N. Sugahara, Andrew M. Lowy

Last updated by author(s): 02/08/2021

Reporting Summary

Nature Research wishes to improve the reproducibility of the work that we publish. This form provides structure for consistency and transparency in reporting. For further information on Nature Research policies, see our Editorial Policies and the Editorial Policy Checklist.

Statistics

For all statistical analyses, confirm that the following items are present in the figure legend, table legend, main text, or Methods section.

n/a	Confirmed	
☐	☒	The exact sample size (n) for each experimental group/condition, given as a discrete number and unit of measurement
☐	☒	A statement on whether measurements were taken from distinct samples or whether the same sample was measured repeatedly
☐	☒	The statistical test(s) used AND whether they are one- or two-sided<br>*Only common tests should be described solely by name; describe more complex techniques in the Methods section.*
☐	☒	A description of all covariates tested
☐	☒	A description of any assumptions or corrections, such as tests of normality and adjustment for multiple comparisons
☐	☒	A full description of the statistical parameters including central tendency (e.g. means) or other basic estimates (e.g. regression coefficient) AND variation (e.g. standard deviation) or associated estimates of uncertainty (e.g. confidence intervals)
☐	☒	For null hypothesis testing, the test statistic (e.g. F, t, r) with confidence intervals, effect sizes, degrees of freedom and P value noted<br>*Give P values as exact values whenever suitable.*
☒	☐	For Bayesian analysis, information on the choice of priors and Markov chain Monte Carlo settings
☒	☐	For hierarchical and complex designs, identification of the appropriate level for tests and full reporting of outcomes
☐	☒	Estimates of effect sizes (e.g. Cohen's d, Pearson's r), indicating how they were calculated

Our web collection on statistics for biologists contains articles on many of the points above.

Software and code

Policy information about availability of computer code

Data collection	For flow cytometry FACS Diva software version 8 was used for data collection<br>For immunofluorescence data collection ZEN software was used
Data analysis	For flow cytometry Flojo version 10 was used for data analysis.<br>For Immunofluorescence quantification Imaje J version 1.47 was used<br>For graphs and statistical analysis Graph Pad Prism 9 was used

For manuscripts utilizing custom algorithms or software that are central to the research but not yet described in published literature, software must be made available to editors and reviewers. We strongly encourage code deposition in a community repository (e.g. GitHub). See the Nature Research guidelines for submitting code & software for further information.

Data

Policy information about availability of data

All manuscripts must include a data availability statement. This statement should provide the following information, where applicable:

- Accession codes, unique identifiers, or web links for publicly available datasets
- A list of figures that have associated raw data
- A description of any restrictions on data availability

The authors declare that all relevant data are included in the paper and its supplementary information files. Source data for figures 1, 2, 3, 4, 5, 6, S5, S7, S9, S11, S14, S15 and S16 are provided with the paper.

Field-specific reporting

Please select the one below that is the best fit for your research. If you are not sure, read the appropriate sections before making your selection.

[x] Life sciences   [ ] Behavioural & social sciences   [ ] Ecological, evolutionary & environmental sciences

For a reference copy of the document with all sections, see nature.com/documents/nr-reporting-summary-flat.pdf

Life sciences study design

All studies must disclose on these points even when the disclosure is negative.

Sample size	Sample sizes for treatment study in Fig 6 were assessed using power calculation at p<0.05, power >0.80 for our initial estimates, but since we had no real preliminary data, the effect size was a bit below projected and so we increased the total N after a planned interim analysis. For the rest of the experiments 3 independent experiments were initially performed, if there was a strong trend and the p value was close to statistical significance 1-2 more experiments were performed.
Data exclusions	One wild type mouse was excluded from the data on radiant efficiency and area of mCherry signals in Fig 5B due to the lack of mCherry signal noted during IVIS Xenogen analysis despite the presence of an mCherry positive tumor confirmed upon necropsy. The lack of mCherry signals during in vivo imaging was likely caused by internal organs overlying the tumor that interrupted with the signals.
Replication	All experiments were confirmed to be reproducible through at least 3 biological replicates.
Randomization	Mice used for the study were randomly allocated into the different treatment groups. All groups had a similar average tumor size before treatment. For the rest of the experiments such as multiwell tissue culture or QPCR, the location in the plate of a given group was changed between different experiments to make sure location had no effect on the results.
Blinding	The survival studies in Fig. 6B were performed in a blinded fashion to the extent possible in collaboration with the animal facility personnel. For the rest of the experiments blinding was not possible since there was only one person performing the experiments.

Reporting for specific materials, systems and methods

We require information from authors about some types of materials, experimental systems and methods used in many studies. Here, indicate whether each material, system or method listed is relevant to your study. If you are not sure if a list item applies to your research, read the appropriate section before selecting a response.

Materials & experimental systems

n/a	Involved in the study	
[ ]	[x]	Antibodies
[ ]	[x]	Eukaryotic cell lines
[x]	[ ]	Palaeontology and archaeology
[ ]	[x]	Animals and other organisms
[x]	[ ]	Human research participants
[x]	[ ]	Clinical data
[x]	[ ]	Dual use research of concern

Methods

n/a	Involved in the study	
[x]	[ ]	ChIP-seq
[ ]	[x]	Flow cytometry
[x]	[ ]	MRI-based neuroimaging

Antibodies

Antibodies used

mouse anti-human αvβ3 (Clone LM609 Cat # MAB1976, EMD Millipore, Billerica, MA)
mouse anti-human αvβ5 (Clone P1F6, Cat # MAB1961 EMD Millipore)
rat anti mouse αv (Clone RMV7, Cat # 50-104-13, Ebioscience)
rabbit anti-human NRP-1 b1b2 developed at SBP
and Alexa 647 or BV421-conjugated mouse anti-human/mouse αvβ5 (clone ALULA Cat # 565836 or 743669, BD)
anti-fluorescein antibody (Cat # A-889, Invitrogen)
anti-FAP (Cat # ABT-11, Millipore Sigma, Temecula, CA)
Cy3-anti-aSMA (Clone 1A4, Cat # C6198, Millipore Sigma)
anti-ERTR7 (Cat # SC-73355, Santa Cruz Biotechnology, Dallas, TX)
rabbit anti-mouse b5 integrin (Cat # 15459, GeneTex, Irvine, CA)
rabbit monoclonal anti-Ki67 (Clone SP6, Cat # ab16667, Abcam)
mouse monoclonal anti-aSMA (Clone 1A4, Cat # ab7817, Abcam)
rat anti-mouse CD31 (Clone MEC 13.3, Cat # 550274, BD Biosciences)
rabbit anti-αvβ5 polyclonal antibody (Cat # bs-1356R, BIOSS, Woburn, MA)
rabbit polyclonal anti-Cleaved caspase 3 antibody (Cat # 9579S, Cell signaling)

	Rabbit IgG Isotype Control (Cat # NB810-56910, Novus)<br>Purified Mouse IgG1 κ Isotype Control (Clone MOPC-21, Cat # 554121 , BD Biosciences)
Validation	-anti-human αvβ3 (Clone LM609) and anti-human αvβ5 (Clone P1F6) were validated for flow cytometry in several human tumor cell lines, as previously published (Ref 24).<br>-rat anti mouse αv (Clone RMV7) was validated for flow cytometry in several mouse tumor cell lines as previously described (Ref 24).<br>-anti-fluorescein antibody, anti-FAP, Cy3-anti-aSMA (Clone 1A4), anti-ERTR7 and the rat anti-mouse CD31 (Clone MEC 13.3) were validated for immunofluorescence using mouse tumor sections (Refs 19, 33, 34, 37), in the case of anti-fluorescein antibody, tumor sections from mice that had been systemically injected with FAM-iRGD were used (Ref 19).<br>-NRP-1 antibody was generated in house at SBP and previously validated for flow cytometry in our previous publication (Ref 24).<br>-rabbit anti-αvβ5 polyclonal antibody from BIOSS was validated for IHC in paraffin mouse tissue in the following reference:<br>Bian, Qin, et al. "Mechanosignaling activation of TGFβ maintains intervertebral disc homeostasis." Bone Research 5 (2017): 17008.<br>We independently validated it in our mouse tumor paraffin sections.<br>-rabbit polyclonal anti-Cleaved caspase 3 antibody was previously validated for immunohistochemistry using mouse paraffin tissue sections, see reference:<br>Riopel M, Seo JB, Bandyopadhyay GK, Li P, Wollam J, Chung H, Jung SR, Murphy A, Wilson M, de Jong R, Patel S, Balakrishna D, Bilakovics J, Fanjul A, Plonowski A, Koh DS, Larson CJ, Olefsky JM, Lee YS. Chronic fractalkine administration improves glucose tolerance and pancreatic endocrine function. J Clin Invest. 2018 Apr 2;128(4):1458-1470.<br>We independently validated it in our mouse tumor paraffin sections.<br>-Alexa 647 or BV421-conjugated mouse anti-human/mouse αvβ5 (clone ALULA) The ALULA clone specifically recognizes αvβ5 integrin and functionally blocks the interaction between αvβ5 and vitronectin. Although raised against mouse αvβ5, the ALULA antibody crossreacts with human αvβ5. For validation see:<br>Lee P, Bax DV, Bilek MM, Weiss AS. A novel cell adhesion region in tropoelastin mediates attachment to integrin αVβ5.. J Biol Chem. 2014; 289(3):1467-77.<br>We have also validated it for flow cytometry in several mouse and human cell lines expressing αvβ5.<br>-rabbit monoclonal anti-Ki67 (Clone SP6) was validated by the manufacturer in a section of formalin-fixed paraffin-embedded mouse spleen tissue. The section was pretreated using heat mediated antigen retrieval with sodium citrate buffer (pH 6.0).<br>We independently validated it in our mouse tumor paraffin sections.<br>-rabbit IgG and mouse IgG1 Isotype controls were previoulay validated by us as Isotype controls for flow cytometry and functional assays (see ref 24).

Eukaryotic cell lines

Policy information about cell lines

Cell line source(s)	hBCF6008 and hBCF6011 CAFs were purchased from Asterand (Detroit, MI)<br>mPCFAA0779 CAFs were established from mice implanted with fresh primary human PDAC tissue.<br>hPC1356, hPCF1424, hPCF1299, and hPCF1444 CAFs were established from fresh surgical specimens of human PDAC tissue.<br>MIA PaCa-2, PANC-1 and PC3 were purchased from ATCC<br>MCF10CA1a cells were obtained from the Miller laboratory. See ref:<br>Malignant MCF10CA1 cell lines derived from premalignant human breast epithelial MCF10AT cells.<br>Santner SJ, Dawson PJ, Tait L, Soule HD, Eliason J, Mohamed AN, Wolman SR, Heppner GH, Miller FR.<br>LM-PmC cells were generated in the Lowy laboratory from liver metastasis of KPC mice and then transduced with a lentivirus expressing mCherry. See ref below:<br>Tseng WW, Winer D, Kenkel JA, Choi O, Shain AH, Pollack JR, French R, Lowy AM, Engleman EG. Development of an orthotopic model of invasive pancreatic cancer in an immunocompetent murine host. Clin Cancer Res. 2010 Jul 15;16(14):3684-95. doi: 10.1158/1078-0432.CCR-09-2384. Epub 2010 Jun 9. PMID: 20534740; PMCID: PMC3085509.
Authentication	Human cell lines were authenticated by the DNA Analysis Core Facility at the Sanford-Burnham-Prebys Medical Discovery Institute (La Jolla, CA) and by Genetica Cell Line Testing (Burlington, NC), and mouse cell lines were authenticated by American Type Culture Collection (Manassas, VA). The method used was STR profiling.
Mycoplasma contamination	All cell lines tested negative for mycoplasma contamination
Commonly misidentified lines<br>(See ICLAC register)	No commonly, misidentified cell lines were used in the study

Animals and other organisms

Policy information about studies involving animals; ARRIVE guidelines recommended for reporting animal research

Laboratory animals	Mice. strains C57Bl6/129 F1 hybrids, Athymic Nude mice, sex males and females, age 6-10 weeks.<br>Mice are kept at a maximum of 5 mice per cage, they have 12h light and dark cycles and are kept at 67 degrees Fahrenheit and 50% humidity.
Wild animals	No wild animals were used in this study

Field-collected samples	No field collected samples were used in this study
Ethics oversight	All animal experiments were performed according to procedures approved by the Animal Research Committees at Sanford-Burnham-Prebys (SBP) Medical Discovery Institute (La Jolla, CA) and University of California San Diego (UCSD, La Jolla, CA).

Note that full information on the approval of the study protocol must also be provided in the manuscript.

Flow Cytometry

Plots

Confirm that:

- [x] The axis labels state the marker and fluorochrome used (e.g. CD4-FITC).
- [x] The axis scales are clearly visible. Include numbers along axes only for bottom left plot of group (a 'group' is an analysis of identical markers).
- [x] All plots are contour plots with outliers or pseudocolor plots.
- [x] A numerical value for number of cells or percentage (with statistics) is provided.

Methodology

Sample preparation	cells cultured in a monolayer or spheroids, trypsinized to form a single cell suspenssion
Instrument	LSR Fortessa
Software	FlowJo version 10
Cell population abundance	Sorting was done to create the mCherry labeled CAF or tumor cell lines with 98% purity of mCherry positive cells
Gating strategy	A Cell Gate was used to distinguish cells from debris in a FSA/SSA plot, then single cells were gated in a SSA/SSW plot, then Live cells were gated in a FSA/DAPI or PI plot.

- [x] Tick this box to confirm that a figure exemplifying the gating strategy is provided in the Supplementary Information.
